# Infectious human adenoviruses are shed in urine even after disappearance of urethral symptoms

**DOI:** 10.1371/journal.pone.0212434

**Published:** 2019-03-06

**Authors:** Nozomu Hanaoka, Shin Ito, Masami Konagaya, Naomi Nojiri, Mitsuru Yasuda, Tsuguto Fujimoto, Takashi Deguchi

**Affiliations:** 1 Infectious Disease Surveillance Center, National Institute of Infectious Diseases, Tokyo, Japan; 2 iClinic, Sendai, Japan; 3 Department of Urology, Graduate School of Medicine, Gifu University, Gifu, Japan; 4 Department of Urology, Kizawa Memorial Hospital, Minokamo, Gifu, Japan; Sechenov First Medical University, RUSSIAN FEDERATION

## Abstract

**Background:**

Urethritis is a common sexually transmitted disease, and human adenoviruses (HAdVs) have been found to be associated with nonchlamydial nongonococcal urethritis. However, the level and viability of HAdV in the urine of patients with urethritis remain unclear.

**Methods:**

Male patients with urethritis and an asymptomatic group were screened using their First-void urine (FVU) for urethritis-related pathogens to identify those with HAdV DNA. FVU and gargle fluid were collected from all patients including from those in the asymptomatic group. A swab of eye discharge was also collected from patients with eye symptoms. The pharyngeal and/ or ocular fluid was also screened only in cases in which FVU was positive for HAdV DNA. HAdVs were isolated using A549 cell lines and typed by sequencing, and viral shedding during 2 years was quantified using real-time PCR. The prevalence of HAdV was assessed in the urethritis and asymptomatic groups, and viral load, isolated HAdV types, and urethral symptoms were compared between the groups.

**Results:**

The positive detection rate of HAdV DNA was significantly higher in the urethritis group than in the asymptomatic group. Of 398 patients with urethritis, HAdV was isolated in all 32 cases (23 cases in which only HAdV DNA was detected with a mean of 2 × 10^9^ copies/mL in urine samples). Of 124 control cases, one had HAdV monoinfection. The most frequently detected HAdV type was 56, followed by types 37 and 64. Regarding the relationship between symptoms and isolated HAdVs, the virus was isolated for up to 12 days after urethritis symptoms disappeared.

**Conclusions:**

HAdVs were significantly detected and isolated from the FVU of patients with urethritis. Furthermore, high levels of infectious HAdVs are excreted in urine for a long period even after urethritis symptoms disappear.

## Introduction

Sexually transmitted infections (STIs) are a worldwide problem associated with various sexual practices in modern society. Male urethritis is a particularly common STI [1]. To date, several large-scale studies have reported microorganisms involved in urethritis, including *Chlamydia trachomatis* (CT) and *Neisseria gonorrhoeae* (NG), which together account for 60%–70% of all incidences of urethritis [2–6]. Additionally, several other microorganisms have been reported in nonchlamydial nongonococcal urethritis (NCNGU), including *Mycoplasma genitalium* (MG), *M*. *hominis* (MH), *N*. *meningitidis* (NM), *Ureaplasma urealyticum* (UU), *U*. *parvum* (UP), *Haemophilus influenzae* (HI), human adenoviruses (HAdVs), herpes simplex virus (HSV), *Streptococcus pneumoniae* (SP), and *Trichomonas vaginalis* (TV). However, the transmission mechanisms and clinical characteristics of these causative agents remain unclear [1–3,5]. Recently, several epidemiological and case–control studies focusing on urethritis caused by HAdVs have been reported [2–8], but details regarding pathogenicity and clinical presentation are insufficient.

Previously, we conducted a comprehensive investigation of pathogens in approximately 400 patients with urethritis [5]. The targets of investigation were 12 microorganisms with potential involvement in urethritis (NG, CT, MG, MH, UP, UU, HI, NM, SP, TV, HAdVs, and HSV). HAdV-associated urethritis was identified in 16.2% of patients with NCNGU. Of all patients with urethritis, 2.8% had only HAdV infections and 4% had coinfections with UP [5]. HAdVs were detected in 2%–6.6% of patients with urethritis [2,3,5,6]. The characteristics of HAdV- associated urethritis are significantly different from those of CT-positive nongonococcal urethritis: complaints of more intense dysuria than of urethral irritation, reports of less urethral discharge, scant urethral discharge on genital examination that more often was serous, frequent observation of meatitis and/or balanitis, and more complaints of conjunctivitis symptoms [2,4,5].

Several studies have reported HAdVs in the urine of patients with urethritis, including type 11 of HAdV-B species and types 8, 19, 37, 56, and 64 of HAdV-D species [4,9–15]. HAdV is a DNA virus that primarily infects the respiratory organs, eyes, and digestive tract. It is classified into seven species on the basis of disease and clinical feature [International Committee on Taxonomy of Viruses; ICTV: https://talk.ictvonline.org/], and >80 serotypes and genotypes have been reported to date [Human Adenovirus Working Group: http://hadvwg.gmu.edu/]. The involvement of HAdV in the urinary organs from the first discovery of HAdV and the involvement of HAdV-B type 11 (HAdV-B11) [16] and HAdV-D type 37 (HAdV-D37) [13, 15] have been reported so far. However, much remains unclear about the pathology and mechanisms of pathogenicity of HAdVs. HAdV-B11 is found primarily in hemorrhagic cystitis and has received attention for causing opportunistic infections in patients undergoing kidney transplants and other organ transplants [17–19]. Certain types of HAdVs associated with urethritis are linked to epidemic keratoconjunctivitis (EKC), i.e., HAdV-D [20]. Recently, HAdV-D37, 53, 54, 56, and 64 of EKC-related HAdV-D have been epidemic in Japan [Infectious Agents Surveillance Report; IASR: https://www.niid.go.jp/niid/en/iasr-e.html]. In particular, HAdV-D54 has been identified primarily in Japan and is the most common type of HAdV detected in patients with EKC since 2014 [21,22]. HAdV-D64 was previously designated as HAdV-D19a and retyped using complete genetic analysis [14].

In the present study, to verify whether HAdVs are detected with a statistical significance in the urine of patients with urethritis, we focused on HAdV-associated urethritis in NCNGU cases. We further compared HAdV-positive detection rates between patients with urethritis who underwent STI screenings for >2 years and an asymptomatic group with no subclinical infections caused by related microorganisms. Additionally, to reveal the HAdV types associated with urethritis and virus viability, we conducted a detailed investigation of HAdV types, excretion periods, urethral symptoms, and course of conjunctivitis and pharyngitis symptoms.

## Methods

### Population, sampling, and data collection

Patients with acute urethritis were enrolled at the first medical examination at a urology clinic (iClinic, Sendai, Japan) between June 2014 and May 2016, as the acute urethritis group. The specimens and patient information of 240 cases reported in previous study [5] between June 2014 and July 2015, were used in this study. First-void urine (FVU) and gargle fluid were collected from all patients and a swab of eye discharge was collected from patients with urethritis with eye symptoms, which was suspended in universal transport medium (Copan Diagnostics, Inc., CA, USA). Patients information included estimated infection day, date, and activity; estimated onset date; sexual orientation; age; and symptoms. The medical observation of symptoms of urethritis, pharyngitis and conjunctivitis was also recorded by physicians.

Healthy male volunteers who had no antimicrobial treatment for at least 1 month, as well as no symptoms of urethritis or any other disease were also enrolled as the asymptomatic group during the same period, and their FVU samples were collected for performing the screening described below. Their purpose for visiting to clinic was health examination including STI check-up (Table 1). All specimens were collected with written informed consent under approved protocols by the Ethical Committee of our institute [5]. Specimens of urine, gargle fluid, and eye swab fluid (if necessary) were collected from all consenting men examined at an STI clinic, stored at 4°C, and tested in the laboratory within 1 week of collection.

**Table 1:** Patient characteristic.

	Acute urethritis group	No symptoms; asymptomatic group
Characteristics	n = 398	n = 163
Sampling period	June 2014—May 2016	
Age: median (range)	31 (26–41)	32 (27–39)
MSM	5 (2 homosexual, 3 bisexual)	4 (1 homosexual, 3 bisexual)
Consultation purpose	Diagnosis and treatment for urethritis	STI checkup
Clinical diagnosis	Acute urethritis	Healthy
Sexual activity ^a^	Yes	
Symptom^b^	Yes	No
Leukocytes in first-void urine	≥5^c^ and/or ≥10^d^	<20/μL

^a^ Within 3 months: Sexual activity including genital sex, oral sex, anal sex and/or masturbation.

^b^ Objective discharge, dysuria and/or urethral irritation and/or meatitis and/or balanitis.

^c^ Leukocytes per high-power field in the gram-stained urethral smear.

^d^ Counted by an automated quantitative urine particle analyzer (UF-1000i; Sysmex Corporation, Kobe, Japan).

Acute urethritis was diagnosed on the basis of complaints of one or more urethral symptoms (discharge, dysuria, and urethral irritation), ≥5 leukocytes per high-power field (×1,000) in the gram-stained urethral smear, and/or pyuria with ≥10 leukocytes per 1 μL FVU counted using an automated quantitative urine particle analyzer (UF-1000i; Sysmex Corporation, Kobe, Japan) [5] and STI guidelines [http://jssti.umin.jp/pdf/guideline-2016.pdf].

### Microorganism detection methods

Newly added specimens were tested for NG, CT, MG, MH, UU, HI, NM, SP, TV, HAdVs and HSV as described previously [5]. Briefly, urethral swabs of all patients were cultured for NG, NM, HI and SP. To detect NG and other microorganisms, FVU specimens were examined using nucleic acid amplification tests. NG and CT were detected using by APTIMA Combo2 (Hologic, Bedford, MA, USA). MG, MH, UP and UU were detected using InvaderPlus assay (LSI Medience Corporation, Tokyo, Japan), and TV and HSV were detected using PCR-based assays as reported previously [5]. Additionally, the gargle fluid and swab of eye discharge were tested using the same method in only the HAdV DNA positive patients. HAdV DNA was detected using both real-time [23] and nested [24] PCR.

Virus isolation was performed as previously described [25]. Briefly, all HAdV DNA positive specimens were inoculated into cultured A549 cells (ATCC CCL-185). HAdV was isolated from FVU, gargle fluid or swab of eye discharge using the A549 cell line maintained in Eagle’s minimal-essential medium supplemented with 5% fetal bovine serum. The culture was incubated for at least 1 month through several cell passages until cytopathic effects (CPEs) were observed. HAdV isolation was confirmed by observation of CPE and detection of viral genome with real-time PCR. HAdV typing [24,26,27] and sequencing using the partial sequences of Hexon [24] and Fiber regions [27] were performed using BLAST analysis (NCBI: https://blast.ncbi.nlm.nih.gov/Blast.cgi) on sequences obtained following the established method. Nucleic acid solution (100 μL) was extracted using the High Pure Viral Nucleic Acid Kit (Roche Diagnostics K.K., Tokyo, Japan) using 200 μL culture medium including CPE cells, urine, gargle fluid, or eye swab solution. The number of HAdV copies was calculated [23]. For specimens in which HAdV DNA was not detected, the number of copies was set at 0 for statistical analysis, but these were excluded from calculations of mean and median values.

### Comparative study

**Fig 1:**
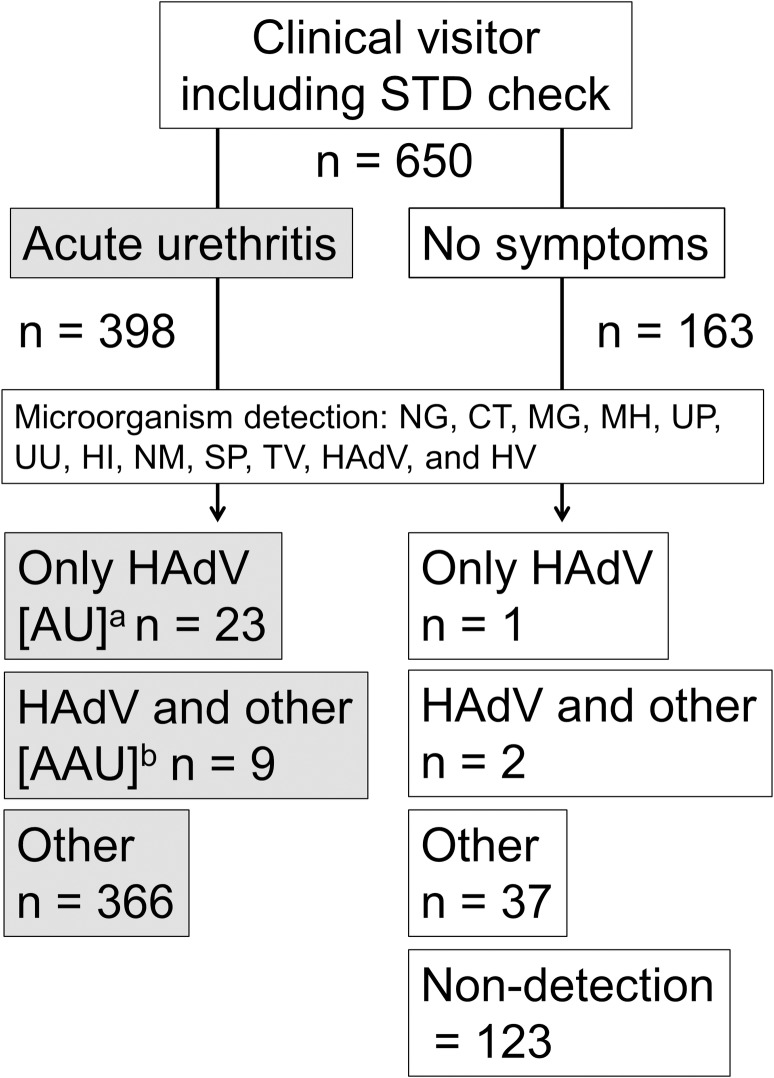
Comparative study flow diagram. A total of 650 clinical visitors were enrolled in this study. They were categorized into two groups. The acute urethritis group included 398 cases, and the no symptoms (asymptomatic) group included 163 individuals. FVU was used for all tests. All specimens underwent screening for the following microorganisms: NG, CT, MG, MH, UP, UU, HI, NM, SP, TV, HAdVs, and HSV. ^a^The 23 cases in which only HAdV DNA was detected were confirmed as adenoviral urethritis (AU) cases and ^b^the nine cases in which HAdV DNA and other pathogens were simultaneously detected were considered as adenovirus-associated urethritis (AAU) cases. A total of 32 cases were confirmed as urethritis with adenovirus in the acute urethritis group. Among the 163 individuals in the asymptomatic group, only one case was positive for HAdV DNA alone and two cases simultaneously had both HAdV DNA and other pathogens. Other pathogens were detected in 37 FVU samples, and 124 patients exhibited no pathogens in urine. Abbreviations: NG, Neisseria gonorrhoeae; CT, Chlamydia trachomatis; MG, Mycoplasma genitalium; MH, Mycoplasma hominis, UP, Ureaplasma parvum; UU, Ureaplasma urealyticum; HI, Haemophilus influenzae; NM, Neisseria meningitidis; SP, Streptococcus pneumoniae; TV, Trichomonas vaginalis; HAdV, Human adenovirus; HV, herpes simplex virus.

All enrolled patients are indicated as clinical visitor in Fig 1 and were categorized into two groups: with acute urethritis and asymptomatic. The acute urethritis group included patients with several symptoms as described above. The asymptomatic group in Fig 1 indicates individuals without symptoms of urethritis, pharyngitis, and conjunctivitis. Patient characteristics are presented in Table 1.

### Statistical analysis

All comparisons were performed with a significance level of *p < 0*.*05*. Statistical analyses were performed using Microsoft Excel 2007 and Excel Toukei ver. 7.0 (ESUMI Co., Ltd., Japan). Fisher’s exact test was used to compare differences in ratios between the urethritis and asymptomatic groups.

### Ethical considerations

This study’s research plan was approved by the ethics review board of the National Institute of Infectious Diseases (No. 586). All specimens were obtained from patients who agreed to participate in the study and signed consent forms.

## Results

### Characteristics of the study population

#### Acute urethritis group

**Fig 2:**
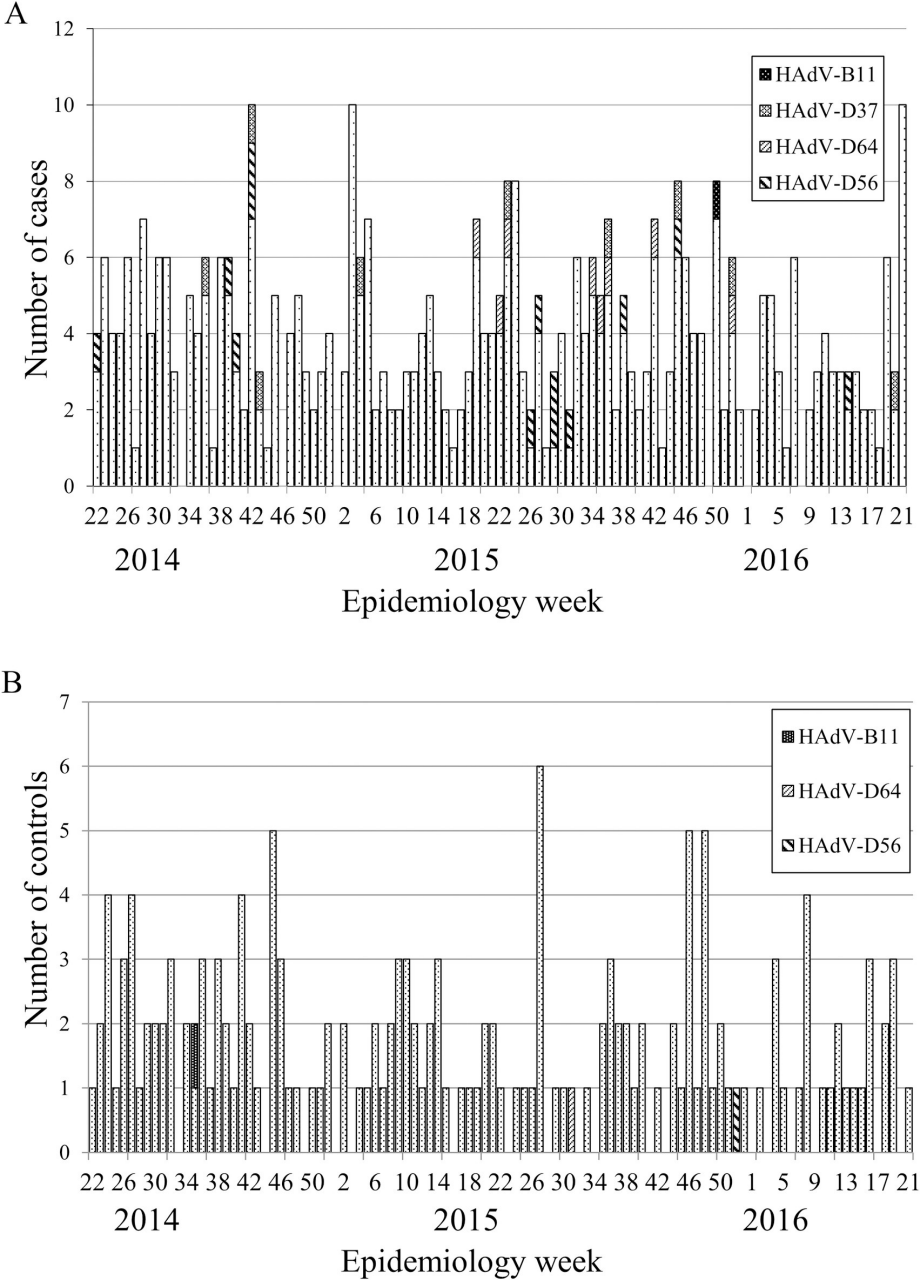
Clinic visitors during the study period. A. The number of acute urethritis cases. The X-axis indicates the epidemiology week, and The Y-axis indicates the number of cases. HAdV DNA detected cases are shown by HAdV types. B. The number of no symptoms cases. The X-axis indicates the epidemiology week, and The Y-axis indicates the number of cases. HAdV DNA detected cases are shown by HAdV types. The number of patients did not differ greatly throughout the years between A and B. No clear seasonal fluctuations in HAdV DNA detection were observed.

We studied 398 patients with urethritis who underwent initial examination at the clinic between June 2014 and May 2016 (Fig 1). Median age (interquartile range) was 31 (26–41) years (Table 1). Fig 2A shows changes in number of cases per month; this number did not vary greatly by month or year. A total of 15 patients had received antibiotics less than 1 month previously. Overall, five patients were men who have sex with men (MSM; two homosexual and three bisexual) (Table 1). The first microbial screening using FVU was for NG and/or CT-associated urethritis, with 165 confirmed cases of NCNGU.

#### Asymptomatic group

We enrolled 163 healthy male volunteers who had no antimicrobial treatment for at least 1 month, no symptoms of urethritis or any other disease, and agreed to participate in the study. Of them, four were MSM (one homosexual and three bisexual) (Table 1). Fig 2B shows the number of volunteers per month, which was not very different from the number of the cases in the urethritis group. Median age (interquartile range) was 32 (27–39) years (Table 1). Thirty-seven urine specimens were positive for urethritis-associated microorganisms (except HAdV), two were positive for HAdV DNA and other pathogens, and only one was positive for HAdV DNA alone. The remaining 123 patients did not exhibit pathogens in urine (Fig 1).

### Characteristics of adenovirus-positive patients with urethritis

Urethritis patients without gonorrhea or chlamydia were considered to have NCNGU. Of these patients, those who tested positive only for HAdV DNA and not for any other urethritis-associated microorganisms were defined as “adenoviral urethritis” (AU; Fig 1). Patients who tested positive for other urethritis-associated microorganisms were defined as “adenovirus-associated urethritis” (AAU; Fig 1). All patients who tested positive for HAdV DNA were defined as having “urethritis with adenovirus.”

Of 398 patients who matched our definition of urethritis and agreed to participate in the study, 165 had NCNGU [median age 34 (27–41) years], including 32 with urethritis with adenovirus [median age 38 (30–43) years]. The HAdV DNA detection rate was 7.8% overall and 20% among patients with NCNGU. The AU group included 23 patients (5.6%) who tested positive only for HAdV DNA. S1 Table shows the types and copy numbers of HAdV DNA, days of illness, and other data for the AU group. S2 Table also provides data on conjunctivitis and pharyngitis symptoms that were confirmed by a physician during diagnosis. The AAU group included nine patients [median age 35 (28–43) years] who tested positive for HAdV DNA and other microorganisms. S3 Table presents information for all members of this group.

All HAdV-positive patients received sitafloxacin (Daiichi Sankyo Co., Tokyo, Japan) 200 mg/day for 7 days or azithromycin (Pfizer Japan, Inc., Tokyo< Japan) 2 g/day one time at the initial visit. Table 2 summarizes symptoms and HAdV data for patients who underwent multiple examinations.

**Table 2:** Clinical causes of urethritis with adenovirus in cases.

												Microorganisms detected in urine^c^									
Total clinic visits	Case_ID	No. of clinic visits	Days after first specimen collection at first clinic visit (Day 0 means day of first collection)	Days from onset	HAdV type	Virus isolated^a^	Copy number / mL urine	± SD	Urethritis symptoms	Pharyngitis symptoms^b^ / HAdV detected and isolated^a^	Conjunctivitis symptoms^b^/ HAdV detected and isolated^a^	NG	CT	MG	UU	MH	HI	NM	SP	TV	HS
2	092		0	1	37	+	2 × 10^8^	1 × 10^7^	+	- / +	+ / NT	-	-	-	-	-	-	-	-	-	-
	092	2	8	9	37	+	7 × 10^3^	3 × 10^3^	+	- / +	+ / NT	-	-	-	-	-	-	-	-	-	-
																				
	144		0	4	37	+	2 × 10^7^	6 × 10^5^	+	- / -	- / NT	-	-	-	-	+	-	-	-	-	-
	144	2	16	20	37	-	2 × 10^4^	8 × 10^2^	-	- / NT	- / NT	-	-	-	-	+	-	-	-	-	-
																				
	371		0	2	64	+	3 × 10^8^	8 × 10^7^	+	- / -	+ / +	-	-	-	-	-	-	-	-	-	-
	371	2	10	12	64	-	6 × 10^5^	4 × 10^4^	-	- / NT	- / NT	-	-	-	-	-		-	-	-	-
																				
	544		0	5	37	+	1 × 10^6^	7 × 10^4^	+	- / -	- / NT	-	-	-	-	-	-	-	+	-	-
	544	2	5	10	37	+	9 × 10^5^	4× 10^4^	-	- / NT	+ / +	-	-	-	-	-	-	-	-	-	-
3	114		0	1	56	+	6 × 10^9^	6 × 10^8^	+	- / -	- / NT	-	-	-	-	-	-	-	-	-	-
	114	2	7	8	56	+	7 × 10^6^	5 × 10^5^	+	- / NT	- / NT	-	-	-	-	-	-	-	-	-	-
	114	3	28	29	56	+	6 × 10^4^	3 × 10^4^	-	- / NT	- / NT	-	-	-	-	-	-	-	-	-	-
																				
	131		0	4	56	+	5 × 10^7^	2 × 10^7^	+	- / -	- / NT	-	-	-	-	-	-	-	-	-	-
	131	2	9	13	56	+	8 × 10^4^	2 × 10^4^	-	- / NT	- / NT	-	-	-	-	-	-	-	-	-	-
	131	3	21	25	56	+	6 × 10^3^	1 × 10^3^	-	- / NT	- / NT	-	-	-	-	-	-	-	-	-	-
																				
	141		0	3	56	+	2 × 10^6^	2 × 10^5^	+	- / -	+ / NT	-	-	-	-	-	-	-	-	-	-
	141	2	21	24		-	-	-	-	- / NT	- / NT	-	-	-	-	-	-	-	-	-	-
	141	3	35	38	NT	NT	NT	NT	-	- / NT	- / NT	NT	NT	NT	NT	NT	NT	NT	NT	NT	NT
																				
	313		0	12	64	+	7 × 10^6^	9 × 10^5^	+	- / +	+ / +	-	-	-	-	-	-	-	-	-	-
	313	2	14	26	‐	-	-	-	-	- / -	- / NT	-	-	-	-	-	-	-	-	-	-
	313	3	29	41	‐	-	-	-	-	- / -	- / NT	NT	NT	NT	NT	NT	NT	NT	NT	NT	NT
																				
	394		0	7	56	+	2 × 10^5^	2 × 10^3^	+	- / +	+ / +	-	-	-	-	-	-	-	-	-	-
	394	2	9	16	‐	‐	-	-	-	- / +	+ / +	-	-	-	-	-	-	-	-	-	-
	394	3	24	31	‐	‐	-	-	-	- / -	- / NT	NT	NT	NT	NT	NT	NT	NT	NT	NT	NT
																				
	302		0	3	64	+	2 × 10^6^	2 × 10^5^	+	+ / -	- / NT	-	-	-	-	-	-	-	-	-	-
	302	2	10	13	-	-	-	-	+	+ / -	- / NT	-	-	-	-	-	-	-	-	-	-
	302	3	24	27	NT	NT	NT	NT	-	- / NT	- / NT	NT	NT	NT	NT	NT	NT	NT	NT	NT	NT
																				
	439		0	5	11	+	1 × 10^5^	3 × 10^4^	+	+ / -	- / NT	+	+	-	-	-	-	-	-	-	-
	439	2	7	12	11	+	2 × 10^4^	4 × 10^3^	-	+ / -	- / NT	-	-	-	-	-	-	-	-	-	-
	439	3	16	21	11	-	8 × 10^3^	1 × 10^3^	-	- / NT	- / NT	-	-	-	-	-	-	-	-	-	-
																				
	467		0	7	64	+	3 × 10^6^	6 × 10^5^	+	+ / -	- / NT	-	-	-	-	-	-	-	-	-	-
	467	2	15	22	64	+	5 × 10^4^	4 × 10^3^	-	- / NT	- / NT	-	-	-	-	-	-	-	-	-	-
	467	3	36	43	-	-	-	-	-	- / NT	- / NT	-	-	-	-	-	-	-	-	-	-
																				
	477		0	5	56	+	4 × 10^7^	8 × 10^6^	+	+ / -	- / NT	-	-	-	-	-	-	-	-	-	-
	477	2	7	12	56	+	5 × 10^6^	3 × 10^5^	+	+ / -	+ / +	-	-	-	-	-	-	-	-	-	-
	477	3	17	22	56	+	5 × 10^4^	3 × 10^3^	-	- / NT	- / NT	-	-	-	-	-	-	-	-	-	-
																				
	479		0	2	56	+	1 × 10^8^	2 × 10^7^	+	+ / +	+ / +	-	-	-	-	-	-	-	-	-	-
	479	2	14	16	56	+	2 × 10^5^	3 × 10^4^	-	- / +	- / NT	-	-	-	-	-	-	-	-	-	-
	479	3	28	30	-	-	-	-	-	- / NT	- / NT	-	-	-	-	-	-	-	-	-	-
																				
	569		0	2	37	+	4 × 10^7^	3 × 10^6^	+	- / -	- / NT	-	-	-	+	-	-	-	-	-	-
	569	2	5	7	37	+	1 × 10^4^	5 × 10^2^	+	- / +	+ / -	-	-	-	-	-	-	-	-	-	-
	569	3	23	25	37	-	9 × 10^3^	5 × 10^1^	-	- / -	- / NT	-	-	-	-	-	-	-	-	-	-
4	208		0	30	37	+	5 × 10^6^	3 × 10^5^	+	- / +	+ / +	-	-	-	-	-	-	-	-	-	-
	208	2	12	42	37	+	1 × 10^4^	4 × 10^3^	-	- / +	- / NT	-	-	-	-	-	-	-	-	-	-
	208	3	26	56	-	-	-	-	-	- / +	- / NT	-	-	-	-	-	-	-	-	-	-
	208	4	47	77	-	-	-	-	-	- / NT	- / NT	-	-	-	-	-	-	-	-	-	-
																				
	307		0	5	37	+	3 × 10^7^	3 × 10^6^	+	- / -	- / NT	-	-	-	-	-	-	-	-	-	-
	307	2	7	12	37	+	3 × 10^5^	4 × 10^4^	+	- / NT	- / NT	-	-	-	-	-	-	-	-	-	-
	307	3	23	28	37	+	2 × 10^4^	7 × 10^3^	-	- / NT	- / NT	-	-	-	-	-	-	-	-	-	-
	307	4	42	47	-	-	-	-	-	- / NT	- / NT	-	-	-	-	-	-	-	-	-	-
																				
	387		0	6	64	+	3 × 10^10^	2 × 10^9^	+	- / -	- / NT	-	-	-	-	-	-	-	-	-	-
	387	2	21	27	64	+	1 × 10^4^	2 × 10^3^	-	- / NT	+ / NT	-	-	-	-	-	-	-	-	-	-
	387	3	42	48	-	-	-	-	-	- / NT	- / NT	-	-	-	-	-	-	-	-	-	-
	387	4	56	62	-	-	-	-	-	- / NT	- / NT	-	-	-	-	-	-	-	-	-	-
5	137		0	3	56	+	5 × 10^6^	6 × 10^5^	+	- / -	+ / NT	-	-	-	-	-	-	-	-	-	-
	137	2	11	14	56	+	2 × 10^5^	6 × 10^3^	+	- / NT	- / NT	-	-	-	-	-	-	-	-	-	-
	137	3	25	28	NT	NT	NT	NT	-	- / NT	- / NT	NT	NT	NT	NT	NT	NT	NT	NT	NT	NT
	137	4	35	38	NT	NT	NT	NT	-	- / NT	- / NT	NT	NT	NT	NT	NT	NT	NT	NT	NT	NT
	137	5	46	49	NT	NT	NT	NT	-	- / NT	- / NT	NT	NT	NT	NT	NT	NT	NT	NT	NT	NT
																				
	375		0	3	64	+	5 × 10^6^	4 × 10^5^	+	- / -	- / NT	-	-	+	+	+	-	-	-	-	-
	375	2	7	10	64	+	2 × 10^5^	3 × 10^4^	-	- / NT	+ / +	-	-	-	-	-	-	-	-	-	-
	375	3	28	31	64	-	2 × 10^2^	4 × 10^1^	-	- / NT	- / -	-	-	-	-	-	-	-	-	-	-
	375	4	42	45	-	-	-	-	-	- / NT	- / NT	-	-	-	-	-	-	-	-	-	-
	375	5	63	66	NT	NT	NT	NT	-	- / NT	- / NT	NT	NT	NT	NT	NT	NT	NT	NT	NT	NT
																				
	119		0	4	56	+	2 × 10^8^	4 × 10^7^	+	- / -	- / NT	-	-	-	-	-	-	-	-	-	-
	119	2	9	13	56	+	2 × 10^5^	2 × 10^4^	-	- / NT	+ / +	-	-	-	-	-	-	-	-	-	-
	119	3	20	24	56	+	2 × 10^4^	3 × 10^3^	+	- / NT	- / NT	-	-	-	-	-	-	-	-	-	-
	119	4	34	38	-	-	-	-	-	- / NT	- / NT	-	-	-	-	-	-	-	-	-	-
	119	5	48	52	NT	NT	NT	NT	-	- / NT	- / NT	NT	NT	NT	NT	NT	NT	NT	NT	NT	NT

^a^ + means adenovirus isolated from specimens with A549 cell;—means adenovirus not isolated.

^b^ + means typical symptoms appeared;—means no symptoms were observed.

^c^ + means detected;—means not detected.

Abbreviations: HAdV, human adenovirus; NT, not tested; SD, standard deviation; NG, Neisseria gonorrhoeae; CT, Chlamydia trachomatis; MG, Mycoplasma genitalium; UU, Ureaplasma urealyticum; MH, Mycoplasma hominis; HI, Haemophilus influenzae; NM, Neisseria meningitidis; SP, Streptococcus pneumoniae; TV, Trichomonas vaginalis; HSV, herpes simplex virus.

### Characteristics of “adenoviral urethritis” (AU) cases

#### Types

The HAdV types detected included 12 cases of HAdV-D56, 6 cases of HAdV-D64 and 5 cases of HAdV-D37. The mean amount of HAdV DNA was 2 × 10^9^ copies/mL urine, with a minimum and maximum of 1 × 10^5^ and 3 × 10^10^ copies/mL for Cases_IDs 337 and 387, respectively (S1 Table). The mean viral DNA copies/mL by type were 5 × 10^7^, 6 × 10^8^, and 5 × 10^9^ for HAdV-D37, HAdV-D56, and HAdV-D64, respectively. HAdV-D64 had the largest and HAdV-D37 the smallest mean number of copies, but no significant differences were observed.

#### Symptoms

Approximately 12 cases (52%) exhibited conjunctivitis symptoms, whereas Case_IDs 005, 302, 433, and 479 showed pharyngitis symptoms, and three of these also exhibited conjunctivitis symptoms (S1 Table). Attempts to isolate and identify HAdV from the pharynxes and conjunctivae of symptomatic cases resulted in the same HAdV types as those isolated from urine S3 Table shows cases with usable data on incubation periods (estimated days from infection to symptom onset). After excluding Case_IDs 005, 307, 344, 415, and 479, 18 cases remained. Mean, median (range), and shortest and longest incubation periods were 10, 9.5 (4.3–12.8), 1, and 21 days, respectively. Median incubation period was the longest for HAdV-D37 at 11 (7.8–13.5) days, followed by 10 (5–12) and 9 (4–11) days for HAdV-D64 and HAdV-D56, respectively. No significant differences between the types were observed.

Data on the days of illness (from onset to specimen collection) were available for all 23 cases (S1 Table). Mean, median (range), and shortest and longest illness for all HAdV-associated urethritis patients were 6, 4 (3–6.5), 1, and 30 days, respectively. HAdV-D37 had the shortest illness (median, 3; range 2.5–9.8 days), followed by HAdV-D56 and HAdV-D64 [4 (2.8–6.3] and 4.5 (3–6.8] days, respectively]. No significant differences between the types were observed.

Mean, median (range), and shortest and longest course (incubation period + days of illness) of AU cases were 16, 13 (9.5–20.3), 3, and 43 days, respectively (S1 Table). HAdV-D37 had the longest course (median, 15; range 10.3–24.3 days) followed by HAdV-D56 and HAdV-D64 [12 (9–21) and 14 (12–18) days, respectively]. No significant differences between the types were observed. Case_ID 208 had the longest course of illness (S1 Table). Excluding this case, the mean incubation period, days of illness, and days of the course for HAdV-associated urethritis were 9 (4–12), 4 (3–6), and 12 (9–18) days, respectively (S1 Table).

### Characteristics of “adenovirus-associated urethritis” (AAU) cases

Among the AAU group, Case_ID 375 had coinfection of HAdV-D64 with MG, UU, and MH (S3 Table); Case_ID 286 had coinfection of HAdV-D64 with UU and MH; Case_ID 345 and 386 had HAdV-D37; Case_ID 569 had coinfection of HAdV-D56 with UU; Case_ID 144 had coinfection of HAdV-D37 with MH; Case_ID 427 had coinfection of HAdV-D56 with UU and SP; and Case_ID 544 had coinfection of HAdV-D37 with SP. CT- and NG-positive specimens were positive for HAdV-B11. Overall, the mean number of viral copies was 2.7 × 10^7^ copies/mL, which was marginally lower than that for HAdV-associated urethritis (not statistically significant). Except for HAdV-B11, there were no differences in isolated viral strains with HAdV-associated urethritis. We did not compare days of illness or other items owing to the small number of cases.

### Comparative study

The comparative study was performed with prevalence of HAdV DNA detection rate and copy number of HAdV DNAs using the first specimens from each patient. S4 Table summarizes HAdV DNAs detected in the asymptomatic group. There was only one HAdV-positive case (0.8%) without other pathogens. In the asymptomatic group overall, the mean number of HAdV DNA copies/mL in urine samples was 4 × 10^3^ (4 × 10^3^–3 × 10^4^), which is a very small number compared with that in AU cases. Of the 39 controls positive for certain pathogens, two were HAdV-positive (HAdV-B11and HAdV-D64) and exhibited small numbers of viral copies: 3 × 10^3^ and 4 × 10^3^ copies/mL urine, respectively. Both were coinfections with UU and MH (S4 Table). Fisher’s exact test analysis revealed that HAdV DNA was detected significantly more often in the urethritis group (32/398) than in the asymptomatic group [full group, 3/163 (*P* < 0.005); group with no pathogen excluding HAdV, 1/124 (*P* < 0.005)]. The odds ratio (OR) with 95% confidence interval (CI) of HAdV DNA detection rate was also calculated between the urethritis and asymptomatic groups (full group), which resulted in an OR of 4.7 and a 95% CI of 1.4–15.4 (*P < 0*.*01)*.

### Urethritis with adenovirus case results, symptoms, and viral isolation status

Table 2 summarizes the cases of urethritis with HAdV that were reexamined and had courses that could be followed. Of the 32 cases, the courses of 21 cases (70%) could be examined in two or more visits. Case_ID 375 had the longest follow-up, with records showing the presence or absence of symptoms 63 days after the initial visit. The longest period from initial visit to final test was 56 days in Case_ID 387 (Table 2). Three cases were examined five times (the most frequent examinations), whereas 19 were followed until urethritis symptoms disappeared. In 14 of the 21 cases, urethritis symptoms were not observed at the second visit (mean days of illness 19, median 16). In six cases, urethritis symptoms disappeared by the third visit (mean days of illness 26, median 26). Overall, symptoms disappeared by mean (median) day of illness 21 (22).

**Fig 3:**
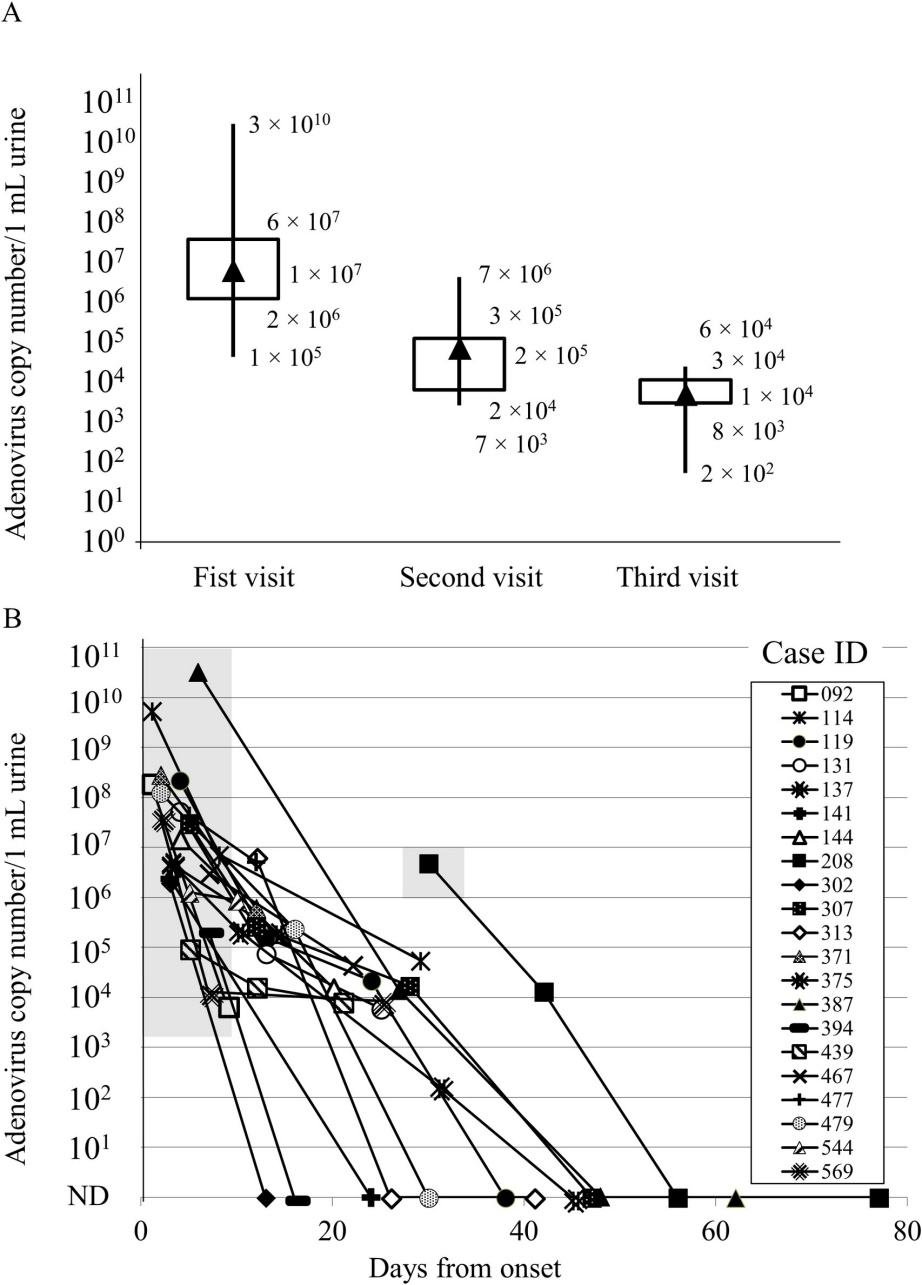
Changes in the number of HAdV copies over time. A. Changes in the HAdV genome copy number at the initial, second, and third visits. Cases in which no virus was detected were excluded. The upper panel shows the maximum, 1st interquartile (25%), 2nd interquartile median (50%, black triangle), 3rd interquartile (75%), and minimum values for the number of viral copies. The Y-axis indicates the number of viral copies per 1 mL of urine. B. Number of viral DNA copies at estimated number of days from onset. Changes in virus levels in cases that could be tested are indicated by solid lines. ND indicates not detected. Gray area shows the period during which symptoms were present. Symptoms were observed from days 1 to 10 in all cases, except for Case_IDs 208 and 544. On day 11, only Case_IDs 307 and 477 were symptomatic. Case_ID 208 was unusual but similar to the others in that the patient was symptomatic at the first visit. As time passed, the viral levels decreased and symptoms disappeared. The X-axis indicates the estimated days since onset. The Y-axis indicates the number of viral copies per 1 mL of urine.

In Case_ID 119, urethritis recurred at the third visit after the symptoms had disappeared at the second visit; therefore, this case was excluded from the analysis. In addition, five cases exhibited pharyngitis at the initial visit and conjunctivitis was also observed in 13. In particular, conjunctivitis occurred in significantly more cases in the AU than in the AAU groups (*P* < 0.05). The pharyngitis and conjunctivitis symptoms disappeared at roughly the same time as the urethritis symptoms. However, in some cases, such as Case_ID 394, conjunctivitis symptoms lasted longer. Four cases had three symptoms (urethritis, conjunctivitis, and pharyngitis). The longest duration of confirmed urethritis symptoms was 11 days (Case_ID 137). Interestingly, five cases without conjunctivitis symptoms at the initial visit demonstrated conjunctivitis at the second visit. S1, S2 and S3 Tables, as well as Fig 3A and 3B summarize the number of visits and number of HAdV DNA copies from onset to visit/specimen collection.

Despite the decrease in viral DNA copies detected over time, substantial levels of HAdV DNA continued to be excreted even after urethritis symptoms ended. Mean number of HAdV DNA copies at the initial visit was 1 × 10^9^ copies/mL urine. At the second visit, the mean (median) number in the seven symptomatic and 12 asymptomatic cases was 2 × 10^5^ (2 × 10^5^) and 2 × 10^4^ (8 × 10^4^) copies/mL urine, respectively. At the last visit, in Case_IDs 141, 313, 394, 302, 467, 179, 208, 307, 387, 375, and 119, the viral copy number decreased to below the detection limit. The asymptomatic group exhibited fewer copies, but the difference was not statistically significant. We also calculated the number of HAdV DNA copies from Cases_IDs 144, 371, 439, 569, and 375, in which the virus had not been isolated and symptoms had disappeared completely. The copy numbers of HAdV DNA in this group were clearly lower than at the initial visit, but significant differences were not observed in comparisons that integrated the presence or absence of symptoms and whether HAdV had been isolated.

### Adenovirus isolation

HAdV was isolated from urine specimens of all cases of urethritis with HAdV at the initial visit and from that of 15 of 21 cases at the second visit. Of these, symptoms persisted in Case_ID 092, 114, 137, 307, 477, and 569, whereas symptoms disappeared in the other nine cases. At the third visit, HAdV continued to be isolated from the urine of Case_ID 114, 119, 131, 307, and 477 but symptoms had disappeared in all of them. At the fourth visit, the virus was not isolated in any case (Table 2). In Case_ID 131, symptoms disappeared at a repeat visit but HAdV was still isolated at a later visit, and the virus continued to be excreted for at least 12 days (Table 2). We compared the number of copies between 15 cases in which HAdV was isolated at the second or later visit and 2 cases in which HAdV could no longer be isolated, and the mean numbers were 1 × 10^6^ (median 2 × 10^5^) and 3 × 10^5^ copies/mL urine, respectively; there was no difference over 10 times. At the third visit, the five cases in which isolation was possible and three cases in which isolation was not possible had a mean (median) of 3 × 10^4^ (6 × 10^3^) and 2 × 10^4^ (8 × 10^3^) copies/mL, respectively. Overall, the copy number of HAdV DNA was lower at the third visit than at the second visit, but the difference between cases in which isolation was possible and not possible was not large (Fig 3A). Furthermore, of cases in which isolation was possible, Case_ID 131 exhibited the smallest number of copies at 6 × 10^3^ copies/mL urine. In Case_ID 114 and 131, HAdV-56 was isolated on the days that symptoms disappeared (days 28 and 21, respectively), indicating that HAdV DNA continued to be excreted for more than 20 days. No significant differences were observed between the HAdV types that were isolated. Regarding the reappearance of symptoms, the longest period from symptom disappearance to confirmation that HAdV DNA excretion had stopped is 21 days in Case_ID 208 (real-time PCR was also negative) (Table 2). In all cases with pharyngitis or conjunctivitis at diagnosis, the same HAdV type as in urethritis cases was isolated. HAdV DNA could be identified from the pharynx and urine, even in cases with no pharyngitis symptoms (Table 2). Except for Case_ID 479, HAdV DNA was not detected in the gargle fluid of any patient in the AU group with pharyngitis symptoms, although HAdV DNA was identified from cases without pharyngitis symptoms but with conjunctivitis symptoms (S1 Table). In the AAU group, presence or absence of the virus was consistent with the presence or absence of pharyngitis symptoms (S3 Table).

### Disappearance of urethritis symptoms, number of HAdV copies, and isolation status

The calculation of subjective symptoms as presented in S1 Table showed that the longest period of urethritis symptoms in this study was 30 days after onset. However, this method lacks objectivity, and clinical visits are a more accurate yardstick. Therefore, 11 days was the longest symptomatic period. After urethritis symptoms disappeared, HAdV, which remained isolatable and infectious for a maximum of 12 days, continued to be excreted in urine at 6 × 10^3^ to 8 × 10^4^ copies/mL. Of the copies present, the level of virus that actually is infectious or isolatable can be assessed via differences in viral culture periods. However, we did not perform filtration sterilization of urine during culturing, so the isolating cultures may have been contaminated with mold or bacteria, making CPE observations of unadulterated virus specimens difficult. Therefore, we set a 1-month limit for the isolation and observation period, and based our assessments only on whether isolation was possible during this period. Thus, the culture periods were not uniform. Nevertheless, all samples for which isolation was successful occurred at the second passage, in less than 3 weeks. This roughly matches the previously reported optimal period for isolation [25].

## Discussion

Previous studies had not clarified the level and viability of HAdV in the urine of patients with urethritis. In this study, HAdV was isolated significantly more frequently from patients with urethritis compared with the asymptomatic group. It was observed in several cases that high levels of HAdV capable of causing infection were excreted in urine for a long period even after the disappearance of urethritis symptoms and that infections can be transferred from the urethra to the eyes.

Seasonal fluctuations in HAdV-associated urethritis have been suggested previously [4] but were not observed in our study. Multiple urethritis-associated pathogenic microorganisms have been simultaneously detected in some cases [2,3,5–8]; therefore, we classified cases of urethritis with HAdV on the basis of presence or absence of other pathogens (AU and AAU groups) to differentiate urethritis caused by HAdV (Fig 1). HAdV DNA was detected significantly more frequently in the urethritis group compared with the asymptomatic groups, and the 8% HAdV DNA detection rate was not substantially different from previously reported rates [2,3,5,6]. The number of cases in which HAdV DNA was detected in the urethritis group (32) was significantly greater than in the asymptomatic group. The detection rate was also significantly higher when limited to AU cases but not when including AAU cases. In the asymptomatic group, pathogen detection was believed to indicate subclinical infection. When HAdV DNA was detected alongside other pathogens in the urethritis group, we did not distinguish whether this was a subclinical infection or a significant coinfection. The amount of UU or MG detected correlates with pathogenicity [5]; therefore, quantitative analyses of these bacteria could provide more detailed information on pathogenicity. Regarding age, the 165 patients with NCNGU (median age, 34 years) in the urethritis group were younger than the 32 with urethritis with adenovirus (median age, 38 years). The median ages of the 23 patients with AU and 9 with AAU were 38 and 35 years, respectively. By type, the median ages for HAdV-D37, -D56, and -D64 were 39, 35, and 30 years, respectively, exhibiting age differences according to types, however, these differences were not significant. The small number of study participants is one reason why significant differences according to age could not be confirmed.

Differences in sexual orientation were not examined because of the small number of MSM (five patients, 1.2% of the total). Although we could not perform comparisons owing to the small number of MSM, a previous report that examined MSM and microorganisms that cause urethritis reported no significant differences from those who engaged in heterosexual intercourse [2].

In our examination of days of illness, we did not compare cases in which other pathogens were identified. A previous report observed no major difference in days of illness between HAdV-associated and CT urethritis [5]. Further, it appeared that in many patients, symptoms disappeared after approximately 1 week; because these patients did not return for another examination, they were considered cured.

The main types of HAdV identified in the present study were HAdV-D37, -D56, and -D64, which suggested a strong relationship with EKC. HAdV-D37 is associated with the urinary tract [13,15,28], and HAdV-D56 was the first urethritis-related HAdV reported in Japan [11]. HAdV-D64 recently was retyped from HAdV-19a [14]. This study shows the types of urethritis-associated HAdV-D11, -D37, -D53, -D56, and -D64 that have been reported in Japan. Interestingly, HAdV-D8, which has been reported worldwide [7,8,15], has not been detected in urine in Japan but has been observed in EKC in Japan [IASR; https://www.niid.go.jp/niid/en/iasr-e/865-iasr/7390-449te.html], therefore, detecting HAdV-D8 in our study was a possibility. After HAdV-G52 had been reported, the typing method switched from serotyping to genotyping. Distinguishing between types is known to be difficult owing to neutralization and the presence of short, partial sequences. With the method [24,25,26] used in our study, types B11, D37, D56, and D64 were able to be ascertained. HAdV-B11 is part of the B species, which has long been linked to hemorrhagic cystitis. Its relationship with urethritis is unclear. Due to its low detection rate and underestimation of the influence of coinfected bacteria, it may not be associated with typical HAdV-associated urethritis. Only two cases of HAdV-B11 were detected in our study (a superinfection with NG and CT and a coinfection with UU).

In this study, significant differences between HAdV types could not be confirmed because of the small sample size and large distribution of values, which was because of the separation of urethritis cases into two groups.

Warnings about HAdV in the eyes (adenoviral conjunctivitis) have been issued because it has long been known to spread as a hospital-acquired infection and to cause outbreaks [29]. Problems have also been experienced when subclinical infections with HAdV cause infection during transplantation [30,31]. In the present study, HAdV was isolated from the eyes, pharynx, and urine of asymptomatic patients, which shows these to be part of the cycle of HAdV infection in the environment. Our study demonstrates that high levels (at least over 10^5^ copies / mL of FVU) of isolatable and infectious HAdVs are excreted in urine, which should be considered for prognosis of HAdV-associated urethritis. These findings suggest that HAdVs from urethritis infect the eyes via the hands or other pathways, including sexual activities, resulting in autoinfection.

A similar observation was made in the pharynx, in that isolatable HAdV continued to be excreted for approximately 1 week after symptoms disappeared. The presence of subclinical infection was also confirmed when pharyngitis symptoms were absent, which means that HAdV infections could spread via the pharynx. We observed no clear correlation between symptoms and number of viral copies; however, as time passed after onset, the symptoms disappeared and copy number of HAdV DNA declined. As of December 2018, no effective, general-use drugs have been developed against HAdV. Therefore, only limited symptomatic therapy aimed at relieving pain and resolving inflammation is available when patients are diagnosed with HAdV-associated urethritis. As this study showed, however, coinfections with other pathogenic microorganisms cannot be ruled out when HAdV is detected in urine, therefore antibiotics may need to be administered. In fact, all patients in this study received antibiotics, either to prevent a secondary bacterial infection or because they were requested by the patient. The symptoms of HAdV-associated urethritis disappeared after approximately 1 week, but we could not determine whether this was affected by the administration of antibiotics.

HAdV-D54 is a HAdV type related to EKC, which is an epidemic in Japan [21,22]. During this study, HAdV-D54 was not detected in any patients with urethritis. Host specificity and other characteristics particular to type D54 have not been discovered. Our results could be highly significant as they relate to investigation of the pathogenicity of HAdV-D54. However, HAdV was not detected, despite the presence of pharyngitis symptoms in some cases, such as Case_ID 302. Further studies should be conducted to clarify the pathology of urethritis, including its symptoms, causative viruses and bacteria, as well as host-related factors.

## Limitations

The presence of other pathogens that were not tested for cannot be ruled out. However, this and previous studies have demonstrated the probable existence of HAdV-associated urethritis with epidemiological significance. Moreover, all patients in whom HAdV DNA was detected received antibiotics at the initial visit, and this is believed to have not influenced the results. In this study HAdVs were typed by partial sequences of Hexon and Fiber regions. In the future, complete genome sequence is required to identify new genotypes, and complete genome sequences of HAdVs should be determined for the isolates of the HAdVs in this study. Regarding follow-up visit, we were supposed to revisit patients as routine medical practice, but we did not make a definite promise for this. Because the re-examination depends on the judgment of the patient, Table 2 is constructed with only those patients who revisited our clinic.

## Conclusions

Over 2 years, we conducted a comparative study that involved patients with urethritis and an asymptomatic group. HAdV DNA was identified significantly more frequently in the acute urethritis group. The major HAdV types detected were HAdV-D37, HAdV-D56, and HAdV-D64, which are the types observed in EKC. Moreover, we found that infectious HAdV continued to be excreted in urine long after the symptoms of HAdV-associated urethritis disappeared. Our results also suggest that infections occur through sexual activity and autoinfection from the urinary tract to the eyes.

These findings indicate that, although HAdV-associated urethritis has a positive prognosis, caution is recommended for approximately 1 month after onset because the infection could spread to the eyes, pharynx, or other people.

## Supporting information

S1 Table Virus type, load, and symptoms in AU; adenoviral urethritis. (XLSX)

S2 Table Summary of all urethritis types, including NCNGU and HAdV types. (XLSX)

S3 Table Virus type, load, and symptoms in AAU; adenovirus-associated urethritis. (XLSX)

S4 Table Cases in no symptom group with detected adenovirus. (XLSX)

## Abbreviations

AAU: Adenovirus Associated Urethritis
AU: Adenoviral Urethritis
CNGU: chlamydial nongonococcal urethritis
CPEs: cytopathic effect
CSW: commercial sex worker
CT: Chlamydia trachomatis
EKC: Epidemic keratoconjunctivitis
FVU: first-voided urine
GU: gonococcal urethritis
HAdVs: Human mastadenoviruses
HI: Haemophilus influenzae
HSV: Herpes simplex virus
MG: Mycoplasma genitalium
MH: Mycoplasma hominis
MSM: men who have sex with men
NCNGU: nonchlamydial nongonococcal urethritis
NG: Neisseria gonorrhoeae
NGU: nongonococcal urethritis
NM: Neisseria meningitidis
PCR: Polymerase chain reaction
SP: Streptococcus pneumoniae
STI: Sexually transmitted infections
TV: Trichomonas vaginalis
UP: Ureaplasma parvum
UU: Ureaplasma urealyticum
